# A Randomized Controlled Trial of a Non-pharmacological Intervention for Cancer-Related Dyspnea

**DOI:** 10.3389/fonc.2020.591610

**Published:** 2020-12-01

**Authors:** Patsy Yates, Janet Hardy, Alexandra Clavarino, Kwun M. Fong, Geoffrey Mitchell, Helen Skerman, Vanessa Brunelli, Isabella Zhao

**Affiliations:** ^1^Queensland University of Technology, Cancer & Palliative Outcomes Center, Kelvin Grove, QLD, Australia; ^2^Mater Health Services, Mater Research—The University of Queensland, Brisbane, QLD, Australia; ^3^The University of Queensland, School of Pharmacy, Brisbane, QLD, Australia; ^4^The University of Queensland Thoracic Research Center, The Prince Charles Hospital, Brisbane, QLD, Australia; ^5^The University of Queensland, School of Clinical Medicine, Brisbane, QLD, Australia

**Keywords:** dyspnea, non-pharmacological interventions, nurse-led interventions, randomized controlled trial, anxiety

## Abstract

**Objectives:** To evaluate the efficacy of a brief tailored non-pharmacological intervention comprising breathing retraining and psychosocial support for managing dyspnea in cancer patients.

**Design:** Multicenter, single blinded, parallel group, randomized controlled trial.

**Setting:** Four major public hospitals, Brisbane, Australia.

**Participants:** One hundred and forty four cancer patients, including 81 who received an 8-week tailored intervention and 63 who received standard care.

**Inclusion Criteria:** Diagnosis of small or non-small cell lung cancer, mesothelioma or lung metastases; completed first line therapy for the disease; average dyspnea rating >2 on (0–10) rating scale in past week; anticipated life expectancy ≥3 months.

**Outcomes:** The primary outcome measure was change in “worst” dyspnea at 8 weeks compared to baseline. Secondary outcomes were change in: dyspnea “at best” and “on average”; distress; perceived control over dyspnea; functional status, psychological distress; and use of non-pharmacological interventions to manage dyspnea at 8 weeks relative to baseline.

**Results:** The mean age of participants was 67.9 (SD = 9.6) years. Compared to the control group, the intervention group demonstrated a statistically significant: (i) improvement in average dyspnea from T1(*M* = 4.5, SE = 0.22) to T3 (*M* = 3.6, SE = 0.24) vs. (*M* = 3.8, SE = 0.24) to (*M* = 4.1, SE = 0.26); (ii) greater control over dyspnea from T1 (*M* = 5.7, SE = 0.28) to T3 (*M* = 7.5, SE = 0.31) vs. (*M* = 6.8, SE = 0.32) to (*M* = 6.6, SE = 0.33); and (iii) greater reduction in anxiety from T1 (*M* = 5.4, SE = 0.43) to T3 (*M* = 4.5, SE = 0.45) vs. (*M* = 4.2, SE = 0.49) to (*M* = 4.6, SE = 0.50). This study found no intervention effect for best and worst dyspnea, distress from breathlessness, functional status, and depression over time.

**Conclusions:** This study demonstrates efficacy of tailored non-pharmacological interventions in improving dyspnea on average, control over dyspnea, and anxiety for cancer patients.

**Clinical Trial Registration:** The trial is registered at the Australian New Zealand Clinical Trials Registry (http://www.anzctr.org.au). The registration number is ACTRN12607000087459.

## Introduction

Dyspnea is a common and distressing symptom experienced by many people with advanced cancer. Estimates of the prevalence of dyspnea range from 29 to 74% in adults in palliative care settings, increasing in the last weeks of life (1). Compared to other cancer types, dyspnea is most common and most severe in primary lung cancer patients, affecting 90% of this patient group (2). Causes of dyspnea in advanced disease are complex and multifactorial, including obstructions or restrictions directly related to lung or pleural involvement or its treatments, factors indirectly related to the disease such as infections, anemia, or respiratory muscle weakness from cachexia, and from comorbid conditions that may be unrelated to the primary presenting problem, such as underlying chronic obstructive pulmonary disease or heart failure (3, 4). Treatment for dyspnea in this population has been medically focused and centered on addressing the underlying causes, with radiotherapy, chemotherapy and pharmacological interventions, and the drainage of effusions most commonly being used to achieve some reduction in this symptom (5–7). However, dyspnea is an especially complex symptom to assess and treat in practice, as the threshold of perception varies widely, with the severity of disease not always directly related to the intensity of breathing discomfort (8).

Dyspnea is a subjective experience of breathing discomfort that derives from interactions among multiple physiological, social, and environmental factors, and can induce secondary physiological and behavioral responses (8). For some patients, dyspnea remains unrelieved despite the use of currently available intervention strategies (6). The multidimensional nature of the dyspnea experience suggests a range of non-pharmacological methods used as adjuncts to medical management offers some potential in reducing the impact of the symptom. Systematic reviews have reported benefits of non-pharmacological interventions for dyspnea management (9–12). The systematic review conducted by Zhao and Yates examined the influence of various intervention components, delivery methods, and clinical contexts on outcomes of non-pharmacological interventions for breathlessness management in participants with lung cancer (10). On the basis of the five eligible studies included in this review, it was concluded that participants with better functional status may be more likely to benefit from the interventions, and that multi-component strategies that are tailored to the participants' individual needs are likely to be more effective.

The primary hypothesis for this study was that, compared to participants who receive standard education for managing dyspnea, participants who receive a non-pharmacological intervention for managing dyspnea delivered using evidence-based psycho-educational strategies will report greater improvement in “worst” dyspnea at 8 weeks. A secondary aim of this study was to examine the relative effectiveness of this intervention over time by comparing change in dyspnea “at best” and “on average,” and change in distress caused by dyspnea, as well as change in participant's perceived control over dyspnea, functional status and psychological distress, and use of non-pharmacological interventions to manage dyspnea at 8 weeks.

## Methods

### Trial Design and Participants

The study involved a multicenter, single blind, parallel group, randomized controlled trial conducted in four major public hospitals in Brisbane, Australia. This project was funded by the National Health & Medical Research Council (NHMRC) and registered with Australian New Zealand Clinical Trials Registry (ACTRN12607000087459). Each site was granted ethical approval from its local research ethics committee. Participants who met the following criteria were invited to participate in the study: (1) a diagnosis of small cell or non-small cell lung cancer, mesothelioma or lung metastases; (2) completed first line therapy for the disease; (3) an average dyspnea rating >2 on an 11 point (0–10) numeric rating scale in the past week; and (4) an anticipated life expectancy of at least 3 months. Participants who had cognitive impairment that would prevent them from responding to a survey questionnaire or who had a life expectancy of <3 months at the time of screening were not eligible. Written informed consent was obtained from participants and/or their careers.

### Intervention

**Figure 1:**
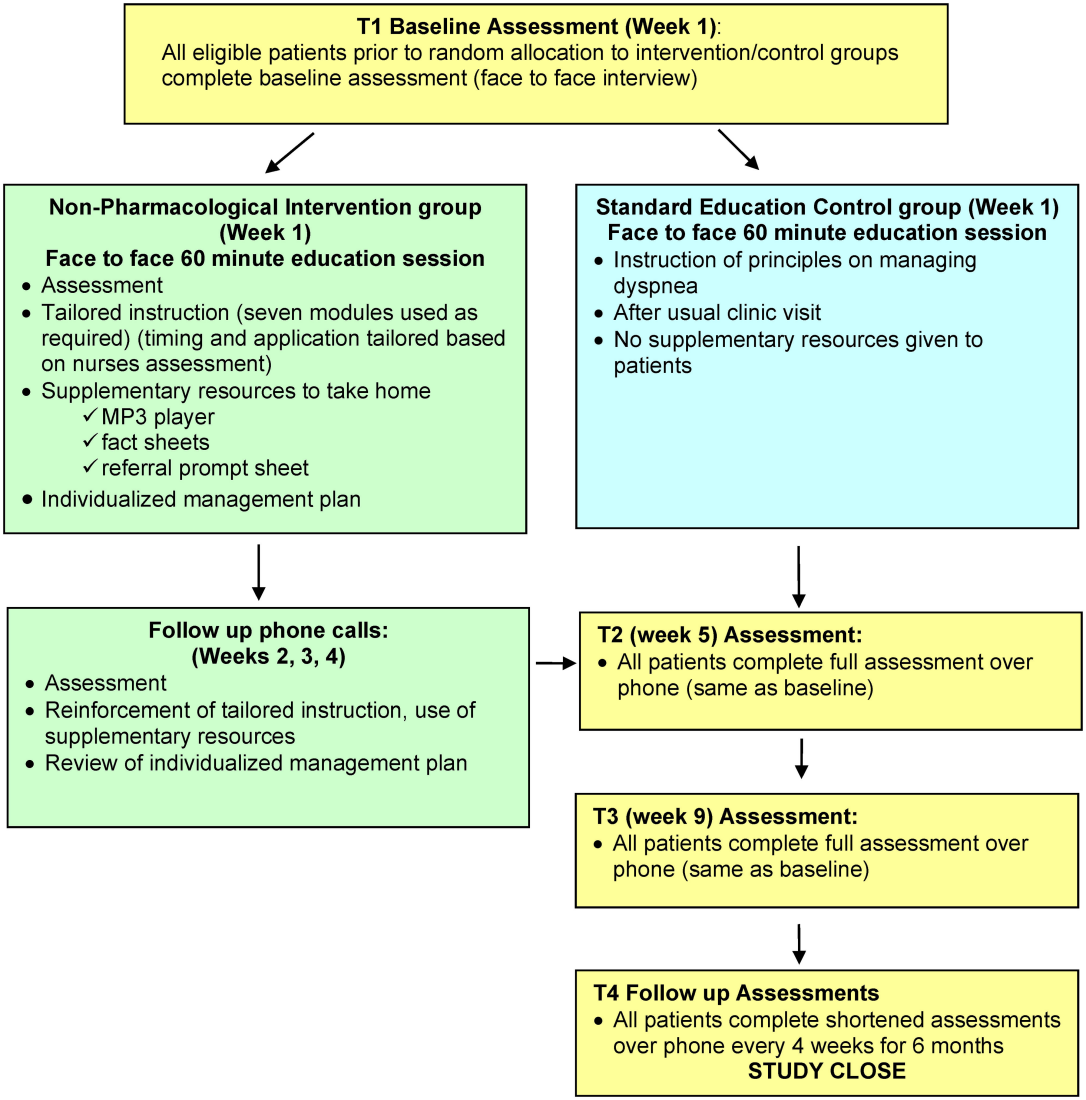
Study protocol.

Participants allocated to the intervention group received a face to face instructional session of about 60 min, followed by weekly phone calls for 3 weeks, to reinforce the strategies (Figure 1). The intervention combined breathing re-training with individualized psychosocial support and was delivered using evidence-based psycho-educational strategies (Box 1). The timing and application of the strategies in the multi-component intervention were tailored to the individual, based on the nurses' assessment, although all components and delivery strategies listed might be used with each participant. The instruction was supplemented by a range of resources to reinforce intervention delivery and promote self-management, including audio recordings, printed fact sheets, an individualized management plan, and a referral prompt sheet. While both groups received education on principles for managing dyspnea, the intervention group differed from the control group in that they also received additional supplementary materials designed to reinforce learning, promote confidence and self-management, and thus enhance intervention outcomes. In addition, participants in both groups continued to receive standard care and other usual supportive care measures, including routine clinic visits, anti-cancer treatments, and other supportive drug therapy or interventions.

Box 1 **Components of the non-pharmacological intervention for dyspnea.**

**Table:** 

**Component**	**Timing**
Detailed assessment of dyspnea its meaning factors that ameliorate or exacerbate it its impact	Week 1—Incorporated into face to face session Weeks 2, 3, 4—Incorporated into telephone follow up
Delivering tailored information on ways of managing dyspnea Instruction incorporates a selection of seven modules on the principles managing dyspnea: Understanding and managing factors contributing to dyspnea Improving breathing efficiency Reducing distress Relaxing Activity pacing Strategies for the caregiver Recognizing when to seek support	Week 1—Face to face session (app. 60 min) delivered by trained nurse Weeks 2, 3, 4—Reinforcement of instruction through telephone follow up sessions (~15 min)
Training in breathing control techniques, progressive muscle relation, and distraction	Week 1—Incorporated into face to face session Weeks 2, 3, 4—Incorporated into telephone follow up
Goal setting to complement breathing and relaxation exercise, to help manage function and social activities Development of an individualized plan documenting: Triggers to breathlessness Specific strategies to be implemented for reducing these triggers, including development of daily activity plans	Week 1—Incorporated into face to face session Weeks 2, 3, 4—Incorporated into telephone follow up
Supporting the family caregiver Involvement of family caregiver where possible in training programs	Week 1—Incorporated into face to face session Weeks 2, 3, 4—Incorporated into telephone follow up
Early recognition of problems warranting medical intervention Prompt sheet for participant and family caregiver to use record referral points, and to facilitate discussion with health care professionals on dyspnea	Week 1—Incorporated into face to face session Weeks 2, 3, 4—Incorporated into telephone follow up

Adapted from Corner et al. (13).

Nurses with experience in working with lung cancer patients were employed to deliver the interventions. The nurses underwent an extensive training program to facilitate a skilled and consistent approach to intervention delivery. The training program included learning activities that aimed to develop advanced knowledge and skills in dyspnea management, supportive communication, and the use of the evidence-based psycho-educational strategies. An intervention protocol was developed to provide the nurse with a framework to tailor intervention techniques to specific dyspnea needs of each participant and to facilitate standardization of the intervention. The quality of the intervention and compliance with study protocols were monitored by investigators who reviewed tape recordings of some sessions selected at random.

### Outcomes

The primary outcome measure of this study was change in “worst” dyspnea at 8 weeks in participants in the intervention group compared to the standard care group. Secondary endpoints were change in dyspnea “at best” and “on average,” and change in distress caused by dyspnea, as well as change in participant's perceived control over dyspnea, functional status, psychological distress, and use of non-pharmacological interventions to manage dyspnea at 8 weeks from the commencement of the intervention, in the intervention group compared to the standard care group. In addition, relevant clinical information was assessed at each time point to enable comparison of intervention and control groups on key clinical and treatment variables that might influence the effectiveness of the intervention or the outcomes of interest to this study.

#### Perceptions of Dyspnea

Five 11-point (0–10) numeric rating scales (NRS) were used to rate dyspnea at best, at worst and on average, distress caused by dyspnea, and control over dyspnea. The NRS has good test-retest reliability (14) and is recognized as an effective measure for patients who are experiencing symptoms such as dyspnea, as it is easily rated by patients who have varying degrees of physical and psychological incapacity (15). One point change on an 11-point numerical rating scale is accepted in recent methodological papers as being a clinically important difference for chronic refractory breathlessness (16).

#### Psychological Distress

Level of psychological distress was assessed using the Hospital Anxiety and Depression Scale (17), which has been widely used as a screening tool for anxiety and depression in cancer patients and has been recommended to be routinely administered to palliative care patients (17, 18). Higher scores indicate higher levels of anxiety and depression. A clinically important difference is indicated by a one-point change on the 11-point numerical rating scale (19).

#### Functional Status

The ECOG Performance Rating scale is widely used to assess how the disease affects the daily living abilities of the patient (20). Scores range from 0 (fully active) to 4 (completely disabled). Functional status was rated by the research nurse from participant responses.

#### Use of Non-pharmacological Interventions

A scale to assess the extent to which participants used the various component strategies was developed in our pilot study. A total of 13 strategies were recommended based on the four modules developed for the non-pharmacological intervention, which could reflect strategies to improve breathing efficiency, reduce distress, relaxation, and activity pacing. Content validity of the items was determined by matching items to components of the intervention, as well as the items included in the breathlessness assessment guide developed in the UK (13). A count was made of the number of recommended non-pharmacological interventions utilized.

### Sample Size

Sample size was calculated using the potential effect size and standard deviation of the primary outcome measure (worst breathlessness) informed by our pilot study (21). In order to detect a 1.6-point mean difference in outcome between groups with a standard deviation of 3.0, a two-sided 5% significance level and 90% power, we required a sample size of 71 participants per group at T3 (8 weeks). Allowing 30% for attrition and 20% for contingencies and potential confounding, the estimated sample size was 214 (107 per arm).

### Randomization

Randomization on a 1:1 basis was by a computer-generated table of random numbers for each site prepared by an investigator with no clinical involvement in the trial. After the research nurse had obtained the participant's consent, a contact independent of the recruitment process at the Institute of Health and Biomedical Innovation (IHBI) at Queensland University of Technology was telephoned for allocation consignment. Participants allocated to the intervention group were aware of the allocated arm, however outcome assessors and data analysts were kept blind to the allocation.

### Statistical Methods

Descriptive statistics and frequency distributions were calculated for participants' demographic and clinical characteristics. The hypotheses were tested using the pooled data from all sites and analyses were done on an intention-to-treat basis. Outcomes were assessed on a priori hypotheses, with each endpoint being considered separately in the analysis. Change in continuous outcome variables over time were examined using Linear Mixed Models (LMMs) and time by group interaction effects. Estimation of the effect of the intervention on breathlessness ratings, anxiety, and depression was based on the mean difference between the intervention and standard care groups at T3 relative to T1. Functional status was coded as a dichotomous variable, so the impact of the intervention on functional status over time was assessed using Generalized Estimating Equations (GEE) and binary logistic regression, assuming an independent correlations matrix. Statistical analyses were performed using SPSS version 18 (SPSS for Windows, Release 18.0; SPSS, Chicago, IL). A 1.6-unit change in breathlessness ratings was considered a clinically important difference. Statistical significance was determined at the conventional level of 5% or less (two-tailed hypothesis tests). Means or odds ratios and 95% confidence intervals (CI) are presented, relevant to the data type. Selected results from these analyses were previously reported in abstract form (22).

## Results

**Figure 2:**
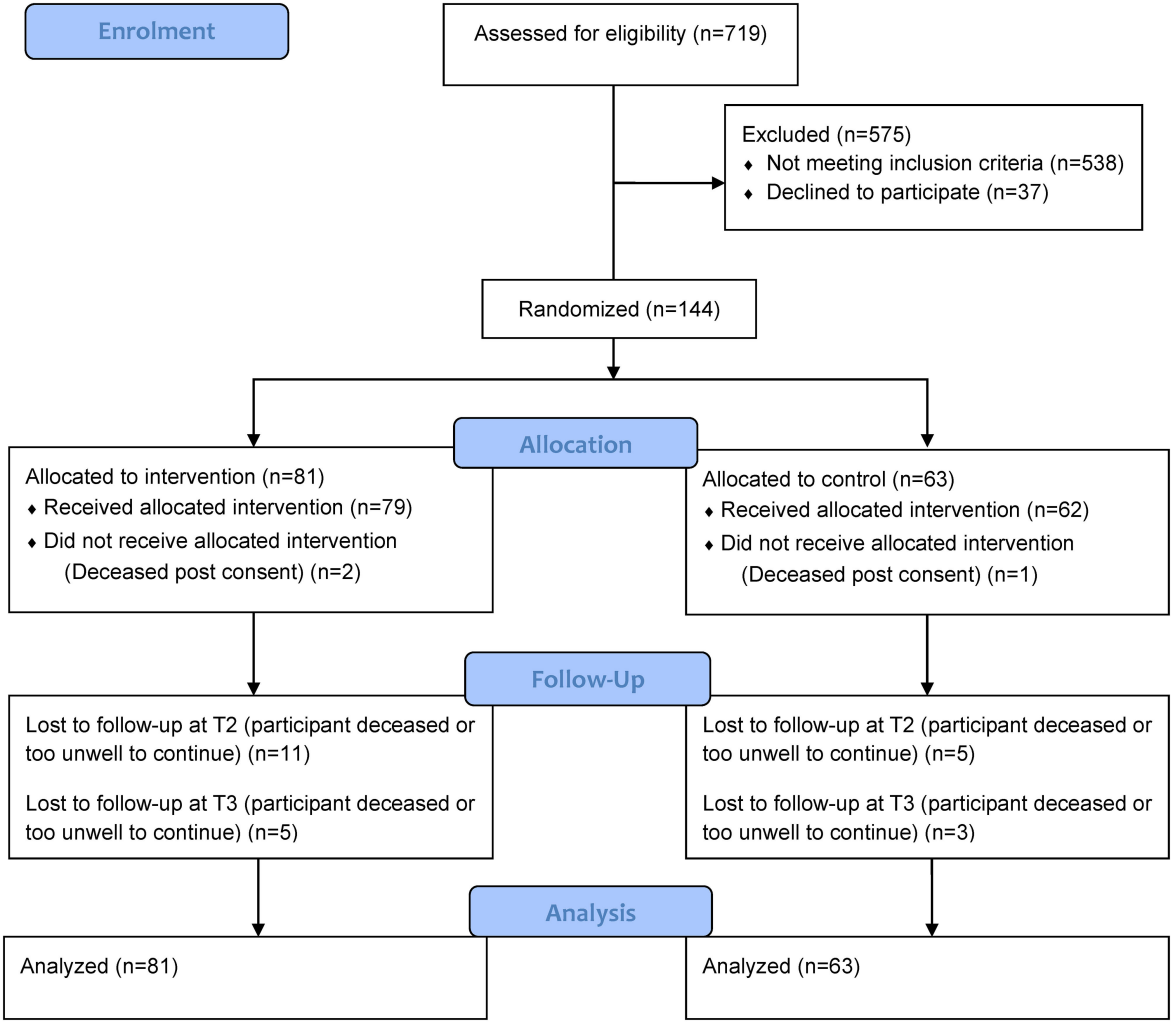
Participant screening and randomization.

From March 2008 through January 2011, a total of 144 participants were recruited at four hospitals and randomized (Figure 2). The attrition rate at 8 weeks was 19% (27 participants); of these, 18 intervention and 9 control participants were lost to follow up. The main reason for withdrawal was that participants were too unwell or deceased. Those lost to follow up did not differ significantly on baseline demographic or medical information from those who remained in the study, except that those lost to follow-up were more likely to have a primary cancer diagnosis of “other” cancers with lung metastases.

Of the 144 participants, 81 were randomly assigned to the intervention group and 63 to the control group. Overall, the mean age was 67.9 (SD = 9.6) years and more than 60% of the participants were male, married or de facto, and lived with a spouse or partner (Table 1). The majority of participants had non-small cell lung cancer as their primary diagnosis (62.6%) and 42.4% of participants had distant metastases at study entry (Table 2). Approximately half of all participants had COPD and one quarter reported having five co-morbid conditions in addition to the primary cancer (Table 2). The proportion of participants who underwent radiotherapy and/or chemotherapy was similar (17.9 and 17.1%, respectively). Baseline demographic, clinical characteristics and medications of the groups are presented in Tables 1, 2.

**TABLE 1:** Demographic information of study participants (*n* = 144).

	**All** **(*****n*** **=** **144)**		**Intervention group** **(*****n*** **=** **81)**		**Control group** **(*****n*** **=** **63)**	
	**Mean** **(SD)**	**No.** **(%)**	**Mean** **(SD)**	**No.** **(%)**	**Mean** **(SD)**	**No.** **(%)**
**Age**^a^	67.9 (9.6)		67.7 (9.1)		68.1 (10.3)	
**Gender**						
Female		53 (36.8)		28 (34.6)		24 (38.1)
Male		91 (63.2)		53 (65.4)		39 (61.9)
**Marital status**^b^						
Single/divorced/separated/widowed		42 (29.2)		23 (28.4)		19 (30.2)
Married/de facto		96 (66.7)		54 (66.7)		42 (66.7)
**Living arrangements**^c^						
Lives alone		31 (21.5)		16 (19.8)		15 (23.8)
With spouse/partner		93 (64.6)		53 (65.4)		40 (63.5)
With children or other		13 (9.0)		7 (8.6)		6 (9.5)
**Highest level of education**^d^						
Did not complete/completed primary school		27 (18.8)		16 (21.6)		11 (17.5)
Completed year 10/certificate		56 (38.9)		29 (35.8)		27 (42.9)
Completed year 12		12 (8.3)		7 (8.6)		5 (7.9)
Vocational training		26 (18.1)		16 (19.8)		10 (15.9)
Tertiary qualification		11 (7.6)		6 (7.4)		5 (7.9)

a n = 131.

b n = 138.

c n = 137.

d *n = 132*.

**TABLE 2:** Baseline medical information of study participants (*n* = 144).

	**All** **(*n* = 144)**	**Intervention** **(*n* = 81)**	**Control** **(*n* = 63)**
**Primary cancer diagnosis**^a^			
Small cell lung cancer	19 (13.2)	11 (13.6)	8 (12.7)
Non-small cell lung cancer	87 (60.4)	47 (58.0)	40 (63.5)
Mesothelioma	13 (9.0)	7 (8.6)	6 (9.5)
Other	20 (13.9)	12 (14.8)	8 (12.7)
**Extent of disease at study entry**^b^			
Localized	30 (20.8)	18 (22.2)	12 (19.0)
Locally advanced	27 (18.8)	16 (19.8)	11 (17.5)
Distant metastases	42 (29.2)	23 (28.4)	19 (30.2)
**COPD**^c^			
Yes	69 (47.9)	40 (49.4)	29 (46.0)
No	70 (48.6)	38 (46.9)	32 (50.8)
**Severity of COPD**^d^			
Mild	20 (29.0)	12 (30.0)	8 (27.6)
Moderate	14 (20.3)	8 (20.0)	6 (20.7)
Severe	21 (30.4)	13 (32.5)	8 (27.6)
**Radiotherapy/chemotherapy**^e^			
Nil	39 (27.1)	17 (21.0)	22 (34.9)
Radiotherapy	25 (17.4)	18 (22.2)	7 (11.1)
Chemotherapy	24 (16.7)	15 (18.5)	9 (14.3)
Both	52 (36.1)	28 (34.6)	24 (38.1)
**Number of co-morbidities**^f^			
0	14 (9.7)	8 (9.9)	6 (9.5)
1	24 (16.7)	10 (12.3)	14 (22.2)
2	22 (15.3)	16 (19.8)	6 (9.5)
3	19 (13.2)	12 (14.8)	7 (11.1)
4	26 (18.1)	14 (17.3)	12 (19.0)
5	35 (24.3)	18 (22.2)	17 (27.0)
**Medication**^g^			
Bronchodilators/anti-spasms	46 (31.9)	24 (29.6)	22 (34.9)
Steroid	27 (18.8)	18 (22.2)	9 (14.3)
NSAIDS	18 (12.5)	10 (12.3)	8 (12.7)
Diuretic	10 (6.9)	5 (6.2)	5 (7.9)
Analgesics	74 (51.4)	41 (50.6)	33 (52.4)
Anti-hypertensive/cardiac drug	69 (47.9)	37 (45.7)	32 (50.8)
Anti-depression/anti-anxiety	55 (38.2)	33 (40.7)	22 (34.9)
Antibiotics	13 (9.0)	5 (6.2)	8 (12.7)
Oxygen	0 (0.0)	0 (0.0)	0 (0.0)
Other respiratory agents	4 (2.8)	2 (2.5)	2 (3.2)
Other drugs	117 (81.3)	66 (81.5)	51 (81.0)

a n = 139.

b n = 99.

c n = 139.

d n = 55.

e n = 140.

f n = 140.

g n = 138.

*All data presented in number (%)*.

### Changes in Dyspnea Severity Ratings Between Groups Over Time

**Figure 3:**
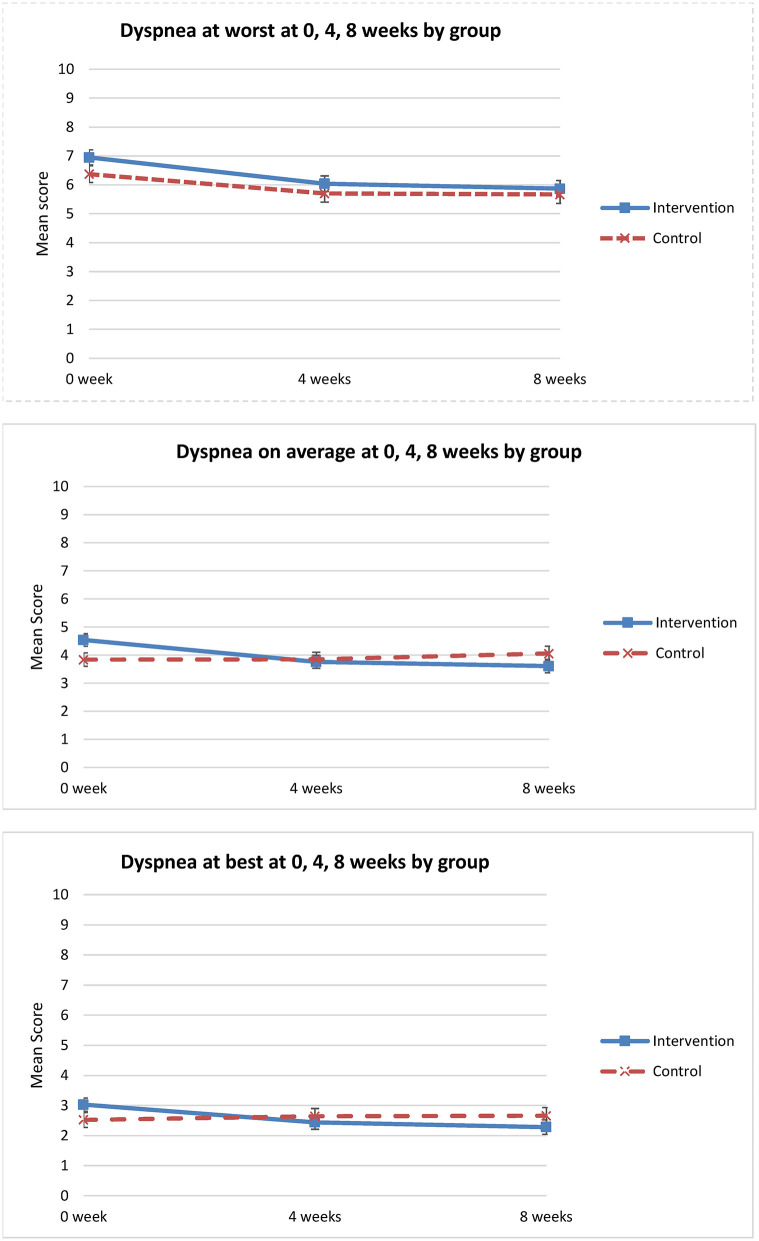
Dyspnea NRS ratings at worst, best and on average over time (0 week, 4 weeks, 8 weeks).

For the primary outcome of worst dyspnea, there was no statistically significant difference in change over time between the groups (*p* = 0.70) (Table 3). Relative to T1, the change in worst dyspnea, indicated by the mean difference between the intervention and control groups was −0.38 (95% CI: −1.30 to 0.53) at 8 weeks (Table 3, Figure 3).

**TABLE 3:** Changes in outcome measures over time by intervention and control groups.

		**Intervention**	**Control**	Group^*^ time	**Intervention**	**Control**		**Effect size**	
		**Mean (SE)**	**Mean (SE)**	***P*-value**	**Mean difference** **(SD)** **relative to T1**	**Mean difference** **(SD)** **relative to T1**	**Mean difference** **(SD)** **relative to T1 and the control group**	**Mean difference between groups/SD**	**95% CI**
Dyspnea at worst	T1	6.95 (0.26)	6.37 (0.29)	0.70					
	T2	6.04 (0.27)	5.70 (0.30)		−0.91 (2.27)	−0.67 (2.27)	−0.23 (2.27)	−0.11	[−0.97; 0.51]
	T3	5.87 (0.28)	5.67 (0.31)		−1.08 (2.27)	−0.7 (2.28)	−0.38 (2.28)	−0.17	[−1.30; 0.53]
Dyspnea on average	T1	4.54 (0.22)	3.84 (0.24)	0.018					
	T2	3.76 (0.23)	3.85 (0.25)		−0.78 (1.93)	0.01 (1.89)	−0.80 (1.91)	−0.41	[−1.48; −0.12]
	T3	3.61 (0.24)	4.06 (0.26)		−0.93 (1.93)	0.22 (1.90)	−1.15 (1.92)	−0.60	[−1.98; −0.31]
Dyspnea at best	T1	3.03 (0.22)	2.52 (0.25)	0.06					
	T2	2.44 (0.23)	2.64 (0.26)		−0.59 (1.93)	0.12 (1.97)	−0.71 (1.95)	−0.36	[−1.41; −0.02]
	T3	2.28 (0.24)	2.66 (0.27)		−0.75 (1.93)	0.14 (1.98)	−0.89 (1.95)	−0.46	[−1.70; −0.08]
Distress caused by dyspnea	T1	4.68 (0.33)	3.39 (0.38)	0.069					
	T2	3.44 (0.36)	2.90 (0.39)		−1.24 (2.95)	−0.49 (2.97)	−0.75 (2.96)	−0.25	[−1.83; 0.32]
	T3	3.04 (0.37)	3.15 (0.40)		−1.64 (2.93)	−0.24 (2.97)	−1.40 (2.95)	−0.47	[−2.60; −0.21]
Control over dyspnea	T1	5.72 (0.28)	6.77 (0.32)	0.001					
	T2	7.06 (0.30)	7.03 (0.33)		1.34 (2.48)	0.26 (2.51)	1.08 (2.49)	0.43	[0.17; 1.99]
	T3	7.49 (0.31)	6.62 (0.33)		1.77 (2.48)	−0.15 (2.48)	1.92 (2.48)	0.78	[0.93; 2.92]
Anxiety	T1	5.39 (0.43)	4.16 (0.49)	0.025					
	T2	4.44 (0.44)	4.30 (0.49)		−0.95 (3.73)	0.14 (3.78)	−1.09 (3.76)	−0.29	[−1.98; −0.21]
	T3	4.51 (0.45)	4.60 (0.50)		−0.88 (3.71)	0.44 (3.77)	−1.32 (3.74)	−0.35	[−2.38; −0.25]
Depression	T1	5.56 (0.42)	5.39 (0.48)	0.202					
	T2	4.99 (0.44)	5.50 (0.49)		−0.57 (3.69)	0.11 (3.74)	−0.68 (3.71)	−0.18	[−1.59; 0.23]
	T3	4.85 (0.45)	5.77 (0.49)		−0.71 (3.66)	0.38 (3.70)	−1.09 (3.68)	−0.30	[−2.34; 0.15]
Number of interventions used	T1	7.37 (0.27)	7.10 (0.31)	0.014					
	T2	8.48 (0.29)	7.15 (0.32)		1.11 (1.81)	0.05 (1.82)	1.06 (1.81)	0.59	[0.74; 1.38]
	T3	8.51 (0.30)	6.99 (0.32)		1.14 (2.39)	−0.11 (2.40)	1.25 (2.40)	0.52	[0.82; 1.68]
ECOG^*^	T1	1.23 (0.09)	1.26 (0.11)	0.537					
	T2	1.24 (0.10)	1.35 (0.11)		0.01 (0.81)	0.09 (0.85)	−0.08 (0.83)	0	
	T3	1.31 (0.10)	1.42 (0.11)		0.08 (0.80)	0.16 (0.84)	−0.08 (0.82)	0	

* *ECOG performance status (16), with higher scores indicating worse performance status*.

**Figure 4:**
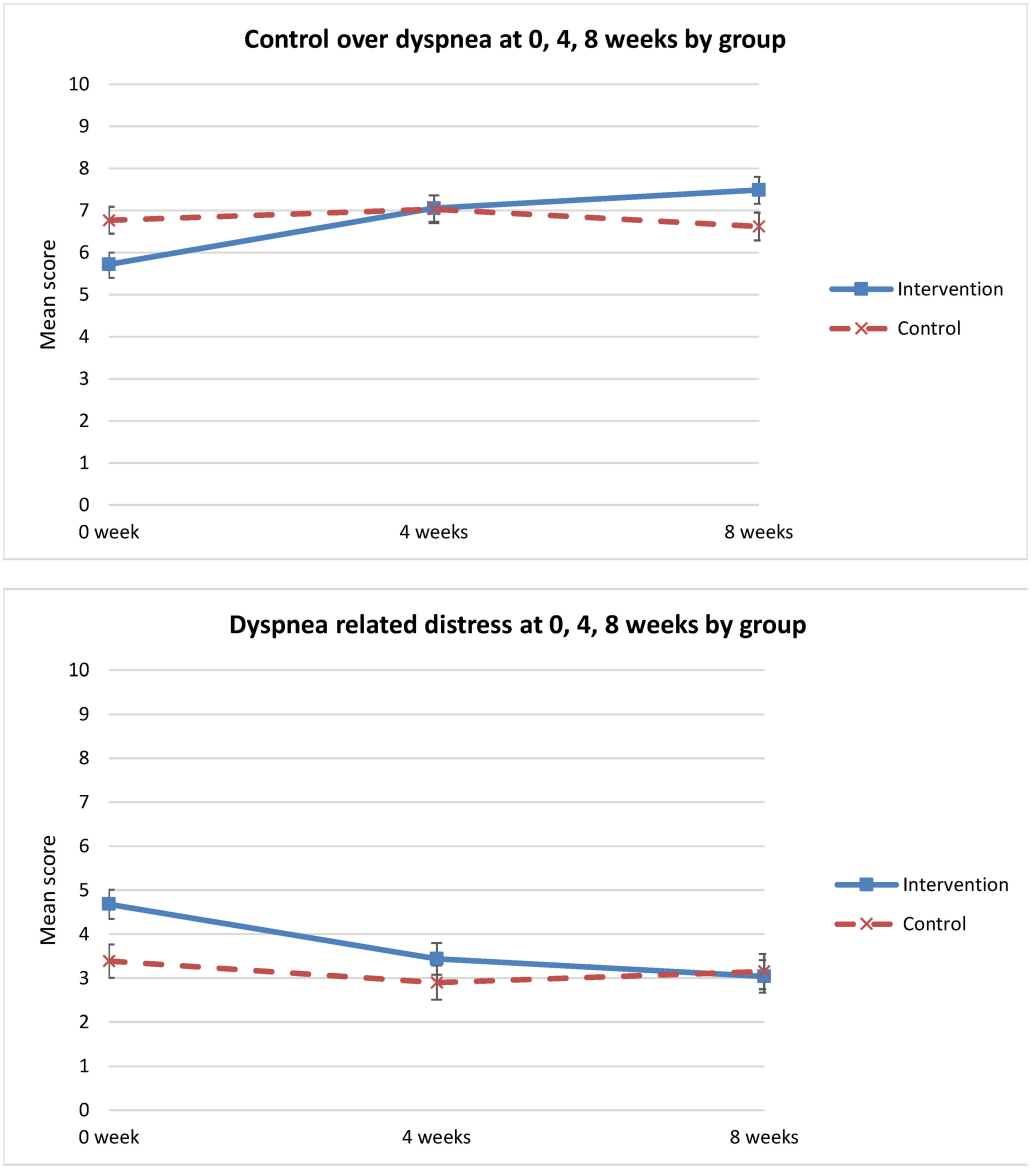
Change in “control over dyspnea” & “dyspnea-related distress” over time (0 week, 4 weeks, 8 weeks).

Analysis of secondary outcomes revealed significant differences between the groups in average dyspnea. Dyspnea on average improved only for the intervention group (*p* = 0.018) (Table 3, Figure 3). Similarly, for other dimensions of the dyspnea experience, significant improvement in perceived control over dyspnea at 8 weeks was observed for the intervention group when compared to the control group (*p* = 0.001) (Table 3). The intervention group perceived increased control over dyspnea from T1 to T3, compared to the control group that showed worsening control over dyspnea from T1 to T3 (Figure 4). Analysis of the other secondary outcomes did not reach statistical significance between the groups over time. For dyspnea at best, the group by time interaction effect was close to significance (*p* = 0.06) (Table 3). In the intervention group, dyspnea at best improved from T1 to T3, but worsened in the control group over time (Figure 3). Similarly, the group by time interaction effect in distress caused by dyspnea was not significant (*p* = 0.07) (Table 3, Figure 4). Relative to T1 and the control group, the greater improvement in dyspnea-related distress in the intervention group at 8 weeks (mean difference = −1.40, 95% CI: −2.60 to −0.21) was statistically significant.

### Change in Anxiety and Depression Between Groups Over Time

**Figure 5:**
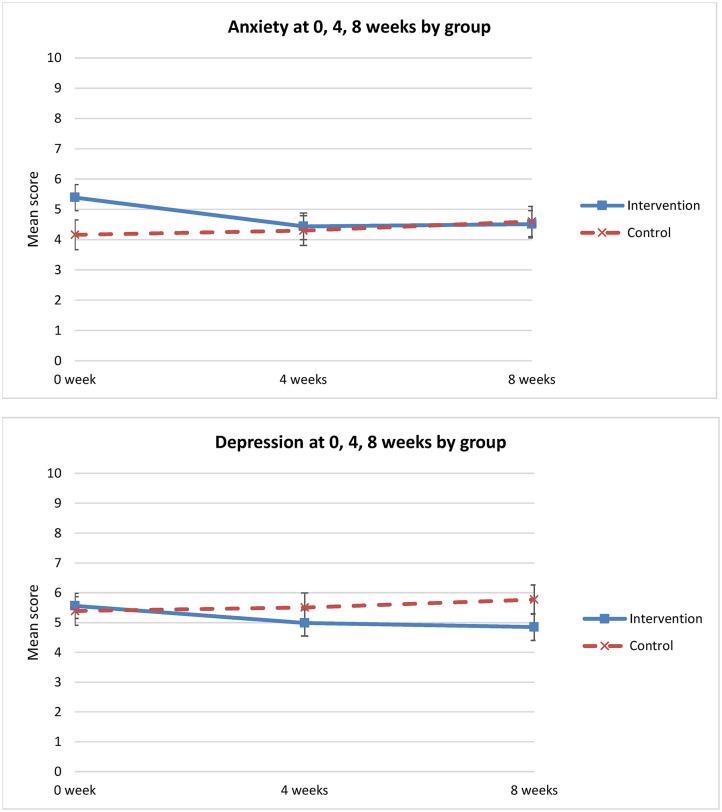
HADS scores over time (0 week, 4 weeks, 8 weeks).

There was a significant change in anxiety between groups and over time (*p* = 0.025). Anxiety level decreased in the intervention group from T1 to T3, but increased in the control group from T1 to T2 and T3 (Figure 5). Relative to T1 and the control group, there was a greater and statistically significant reduction in anxiety in the intervention group at 8 weeks (mean difference = −1.3; 95% CI: −2.38 to −0.25) (Table 3). However, there was no statistically significant difference in depression between the groups over time (*p* = 0.20) (Table 3).

### Change in Functional Status Between Groups Over Time

The two groups did not differ significantly in functional status over time (*p* = 0.41). Relative to T1 and the control group, there was no statistically significant change in functional status in the intervention group at 8 weeks (OR: 0.87; CI [0.53–1.41]) (Table 3).

### Change in Use of Non-pharmacological Interventions Between Groups Over Time

For the number of non-pharmacological interventions used to manage dyspnea, there was a significant difference between groups and over time (*p* = 0.014). Relative to T1 and the control group, the greater use of non-pharmacological interventions by the intervention group at 8 weeks was statistically significant (mean difference = 1.25; 95% CI: 0.82 to 1.68) (Table 3).

## Discussion

Previous studies of non-pharmacological interventions for dyspnea associated with cancer have reported benefits from intensive interventions involving several weeks of face-to-face contact with specially trained health professionals. This multicenter randomized controlled study evaluated a brief tailored non-pharmacological intervention delivered by nurses for managing dyspnea. While no significant effects were demonstrated for the primary outcome “dyspnea at worst,” our findings show that participants receiving the brief self-management focused intervention supplemented by a range of technology enhanced delivery strategies resulted in improvements in dyspnea on average and perceived control over dyspnea, and a reduction in anxiety at 8 weeks, compared to participants receiving standard care. The intervention group also demonstrated increased uptake of the recommended non-pharmacological strategies to manage dyspnea, suggesting the effectiveness of the intervention on reducing breathlessness. On the 0–10 NRS, the levels of improvement in average dyspnea and control over dyspnea at T3 in the intervention group relative to T1 and the control group were more than 1 unit (1.15 and 1.92, respectively). This one point change on an 11-point numerical rating scale is accepted in methodological papers as being a clinically important difference for chronic refractory breathlessness (16). Additionally, the reduction in anxiety level at T3 in the intervention group relative to T1 and the control group was 1.32, indicating clinical significance (19).

We had chosen dyspnea at worst as the primary outcome for this study to be consistent with the initial study of non-pharmacological interventions for breathlessness upon which this intervention was based. While no significant improvement was identified for this primary outcome, the consistent improvements identified for other dyspnea severity measures provide some confidence in the efficacy of this intervention. Indeed, dyspnea on average, is an important indicator of the overall rating of the symptom experienced by the participants, as it takes all situations into consideration and asks the participants to do an overall assessment of dyspnea experienced in the past seven days. As such, our findings reflect important outcomes from the patient's perspective. On the other hand, dyspnea at best and worst is at the extremes of a scale reflecting special and extreme events that only happen rarely. The intervention also did not improve depression compared to standard care. There are a number of predictors for depression in lung cancer patients, including functional impairment, physical symptom burden, and fatigue (23). This brief intervention might not be sufficiently intense to impact this complex symptom, and a more comprehensive approach may be required. The two groups also remained very similar in performance status (ECOG) through the study period. A focus on other concurrent symptoms that impact on functional status, such as fatigue, might be required to have a greater impact on this outcome.

Our results contrast to those of Bredin et al. (24), on which this study was based, who found significant improvement for breathlessness at best, performance status, and levels of depression at 8 weeks in the intervention group (24). There are a number of possible explanations for these differences. Firstly, in Bredin's study, missing data due to the withdrawal of participants from the study were imputed according to a method suggested by Gould (25). This approach has been controversial with the development of multiple imputations; and some studies questioned the validity of the application of multiple imputations (26, 27). Our study did not impute any missing data. We selected LMM as this approach could fully accommodate all of the data available for a subject even if some data were missing (28). Secondly, in Bredin's study, changes in outcome measures between baseline and 8 weeks were calculated and analyzed. This method of analysis assumed that all participants were able to show a change in either direction on the rating scales, as acknowledged by the authors. However, participants whose baseline measurements were at the extremes of a scale would only show change in one direction. From this perspective, LMM is a preferred approach to analyze all three time points, so was selected as the analysis model in our study.

A key focus of our brief intervention was promoting the patient's confidence in self-management of dyspnea. Our findings that patients report a greater sense of control over dyspnea reflect improvement in an important patient-centered outcome. The increased uptake of non-pharmacological interventions reflects the greater confidence in self-management of dyspnea in the intervention group.

### Strengths and Limitations

This multicenter, single blind randomized controlled trial was conducted to evaluate the efficacy of a brief tailored non-pharmacological intervention comprising breathing retraining and psychosocial support for managing dyspnea in lung cancer participants. The success of the nurse led interventions further supports the inclusion of experienced nurses at all stages of care to support participants and carers, as recommended in the most updated National Institute for Health and Clinical Excellence (NICE) guidance (29). The study is novel in that it applies best available evidence about methods for delivering psycho-educational interventions for people with cancer to optimize the delivery of non-pharmacological intervention strategies with proven efficacy. The tailored instructions offered in the first education session were reinforced by telephone calls, weekly for 3 weeks. This reinforcement using flexible health service delivery options is promising. The intervention required minimal clinic time, with different forms of support materials (e.g., booklets, electronic recordings) for participants to use at their own pace and individual situations rather than in a more structured or formal way. The intervention evaluated in this study can be readily incorporated into routine clinical practice to manage the symptom and practitioners could use these guidelines for targeting intervention strategies more appropriately to participants' clinical status and personal goals.

Despite the strengths of this study, the results might not generalize to a wider population. As the participants were recruited from major hospitals in a metropolitan area and the majority of the participants lived in the metropolitan or surrounding areas, their characteristics might be different from those in rural and remote areas. The supplementary take-home materials and telephone follow metropolitan communities.

This is one of the largest randomized controlled trials of non-pharmacological interventions for dyspnea. Despite this, we recorded an imbalance in the number of participants in intervention and control groups most likely due to the use of simple 1:1 randomization at each study site, rather than blocked randomization. The minimum sample size of 71 in each group was not achieved in the control group due to difficulties with recruitment, despite an extended study timeframe. This resulted in slightly <90% power in the analyses. We also did not observe significant differences between groups for the primary outcome, dyspnea at worst. Our targeted sample size was calculated based on change in “worst” dyspnea between groups at week 4 (mean = 1.63, SD = 3.0) in the pilot study (21), which was greater than the mean difference achieved in this trial. The small number of participants in the pilot study (*n* = 30) could have contributed to the larger variation by chance in that study. Despite these statistical limitations, the significant improvements observed across several secondary outcome measures provide some confidence that the intervention has great potential for improving dyspnea management for patients with cancer.

Potential bias should be acknowledged. For example, drop-out bias could have occurred as the attrition rate at 8 weeks was 19%. The main reason for withdrawal was that participants were too unwell or were deceased. However, comparison between the drop-outs and those who remained in the study showed no statistical difference on baseline demographic or medical information, except that those lost to follow-up were more likely to have a primary cancer diagnosis of “other” cancers with lung metastases. Time-related bias should also be considered due to the extended recruitment from March 2008 through January 2011. Despite the time since study completion, the applicability of these findings to contemporary practice remains, given that dyspnea continues to be a significant problem for cancer patients and that no significant advances in non-pharmacological interventions have occurred since this time.

## Conclusion

This multicenter randomized controlled study to evaluate brief tailored non-pharmacological interventions delivered by nurses for managing dyspnea confirm that participants receiving such interventions showed improvement in dyspnea on average, greater control over dyspnea, and a reduction in anxiety over time. The intervention evaluated in this study builds on recent evidence about the importance of tailoring interventions to patient's needs and concerns and demonstrates the value of such approaches in promoting self-management of dyspnea.

## Data Availability Statement

The raw data supporting the conclusions of this article will be made available by the authors, without undue reservation.

## Ethics Statement

The studies involving human participants were reviewed and approved by Queensland University of Technology Human Research Ethics Committee; Mater Health Services Human Research Ethics Committee; Princess Alexandra Hospital Human Research Ethics Committee; The Prince Charles Hospital and Health Service District Human Research Ethics Committee; Royal Brisbane & Women's Hospital Human Research Ethics Committee. The patients/participants provided their written informed consent to participate in this study.

## Author Contributions

PY was the guarantor and has contributed to the planning, conduct, and the reporting of the work. JH, AC, KF, and GM have contributed to the planning and conduct of the work and the review of the manuscript. HS has contributed to the planning, analysis, and reporting of the work. VB has contributed to the conduct of the work and the review of the manuscript. IZ has contributed to the conduct, analysis, and reporting of the work. All authors acknowledge and appreciate the contribution from the study participants.

## Conflict of Interest

The authors declare that the research was conducted in the absence of any commercial or financial relationships that could be construed as a potential conflict of interest.
